# He Taonga Te Wareware: Connecting Older Māori Experiences of Wairuatanga with Mate Wareware (Dementia)

**DOI:** 10.1007/s10823-023-09492-4

**Published:** 2024-01-11

**Authors:** Te Maringi Mai o Hawaiiki, Oliver Menzies, Nick Garrett, Makarena Dudley

**Affiliations:** 1 University of Auckland, Auckland, New Zealand; 2 Te Toka Tumai, Auckland, New Zealand; 3 Auckland University of Technology, Auckland, New Zealand

**Keywords:** Kaumātua, Māori, Mate wareware, Wairuatanga, Spirituality

## Abstract

Mate wareware (dementia) is a complex disease of the brain that progressively inhibits memory and cognitive ability, affecting many Māori (the Indigenous people of Aotearoa New Zealand) kaumātua (elderly persons) in Aotearoa (New Zealand). Mate wareware care aims to protect and sustain wellbeing, yet Māori perspectives of wellbeing that consider wairuatanga (Māori spirituality) are often neglected within current treatment planning. This study investigates the presence of wairuatanga within kaumātua lives, drawing upon 61 interviews with kaumātua to glean a Māori understanding of mate wareware and to develop a diagnostic screening tool for mate wareware. Recorded responses were thematically analysed using reflexive qualitative analysis, informing four key themes that influence wairuatanga: *he hononga tangata* (social connection), *tūrangawaewae* (places of connection), *tuakiritanga* (identity) and *mahi mauritau* (mindful practices). These themes consider the value of creating rich and gratifying lifestyles for kaumātua that cultivate their spiritual wellbeing. This study validates diverse understandings and experiences of wairuatanga as essential to Māori wellbeing, affirming the relevance of wairuatanga to improve outcomes for Māori living with mate wareware.

**Table:** Glossary

Māori words	English translations
Aotearoa	New Zealand
aroha	unconditional love, affection and compassion
he hononga tangata	the connection between people; social connection
he taonga te wareware	forgetfulness is to be treasured
he taunga o te mauri	to familiarise one’s mauri; a place of familiarity and comfort
hōhā	tiresome, wearisome
karakia	prayer; chant
kaumātua	an elderly Māori person; an aged person of status
koha	an offering or gift of thanks
mahi mauritau	actions that nourish wairuatanga
mana	the ultimate and paramount authority of a person derived from the gods
manaaki	to look after, care for others
manaakitanga	selfless care, hospitality and generosity
Māori	an Indigenous person of Aotearoa; the Indigenous ways of Aotearoa
marae	the fenced-in complex of buildings and grounds that serves as a meeting place for iwi, hapū or whānau
mate wareware	dementia; a disease of forgetfulness
mauri	the essential quality and vitality of a living entity; life-force
mokopuna	grandchild; grandchildren
Tāmaki Makaurau	Auckland
Te Tai Tokerau	Northland region
tuakiritanga	the things that shape one’s identity or personality
tūrangawaewae	place where one belongs through rights of kinship and experiences
wairua	Māori spirit; the soul of a person
wairuatanga	Māori spirituality: the enactment of a soul that may manifest in our actions
whakapapa	genealogy; family lines of descendants
whānau	family; the extended, nuanced concept of family

## Introduction

Within Aotearoa (New Zealand), age-related diseases are becoming more relevant as the life expectancy of the population increases (Statistics New Zealand, 2021). Approximately 70,000 people (1.4%) in Aotearoa were living with mate wareware (dementia) in 2020 (Ma’u et al., 2021). Of that number, 4,300 (6.2%) were Māori (the Indigenous people of Aotearoa), although there is speculation that this figure underestimated the actual prevalence in the Māori population (Ma’u et al., 2021; Ministry of Health, 2019). As a result of longer life expectancies, earlier development and higher prevalence of risk factors for mate wareware amongst Māori, it is projected that the prevalence of mate wareware in Māori will triple by 2050 (Cullum et al., 2018; Dudley et al., 2019; Livingston et al., 2020; Ma’u et al., 2021).

Although whānau Māori (Māori family) often conceptualise mate wareware similarly, each whānau also have unique historical, cultural and social contexts which shape their understandings of mate wareware. Within the western world, mate wareware is a complex neurological condition that progressively impairs memory and cognitive ability, reducing everyday functioning, capacity, and quality of life (O’Connor, 2019; Rivera-Rodriguez et al., 2021; Scott & Barrett, 2007). For some Māori, mate wareware may be seen as a normal consequence of growing old and a cherished part of the ageing process (Metge, 1976; Townsend, 2011). The Māori proverbial saying, *he taonga te wareware*, articulates that forgetfulness is a treasure, an enduring act that signifies the wealth of experience and wisdom of one’s life (Dyall, 2014). However, this is not to say that all Māori welcome the symptoms of mate wareware. Some Māori continue to experience significant stigma, fear and shame around the diagnosis of mate wareware and its associated symptoms, most notably due to its nature in reducing a person’s ability to act autonomously according to their identity (Dudley et al., 2019; Townsend, 2011). This shame likely reflects the imposition of western colonial models of wellbeing and individualistic values of independence ascribed upon Māori, dismissing traditional collectivism and Indigenous conceptualisations. Many kaumātua (elderly persons) understand that mate wareware can result from the effects of colonisation, marginalisation, loneliness, the absence of culture or evil misfortune wished upon them (Chamberlain, 2014; Dudley et al., 2019; Dyall, 2014).

Care planning for mate wareware in the health sector lacks a Māori perspective that considers how kaumātua Māori would prefer to be looked after (Moeke-Maxwell et al., 2013). Whānau Māori have expressed concerns about the western monocultural nature of many residential care services that create alienating experiences for kaumātua (Dudley et al., 2019). Due to this estrangement, kaumātua are far less likely to seek residential care and often instead be cared for at home within the whānau environment (Ma’u et al., 2021; Moeke-Maxwell et al., 2013; Menzies et al., 2021). However, there is a risk that whānau carers will not be provided with the appropriate information and knowledge about best care practices by health and disability services (Metge, 1976; Moeke-Maxwell et al., 2008, 2013; Rivera-Rodriguez et al., 2021; Smye & Browne, 2002). When whānau Māori are well-supported and well-informed, they can improve the quality of care provided to kaumātua, prevent the placement of blame on whānau members, and bring understanding, patience and compassion to their caregiving (Tapsell, 2007; Townsend, 2011). Healthcare services must consider Māori approaches to health and, principally, the role of wairuatanga in appropriate Māori-centred care (Bensemann, 2023; Shrestha, 2021).

### Māori Approaches to Health

Māori have unique values, traditions and worldviews that conceptualise health, illness and expressions of distress as holistic and interconnected to all aspects of life (Durie, 1985). Durie’s Te Whare Tapa Model (1985) seeks to illustrate a Māori understanding of wellbeing that considers four interconnected elements of health. He proposes that a person’s wellbeing is determined by the interaction of their physical, mental, social and spiritual health. This spiritual element of wellbeing is known as wairuatanga (Māori spirituality) (Durie, 1985).

Many Māori have unique and personal understandings of wairuatanga that range across regions, tribes and people. Although it is challenging to construct a finite definition of wairuatanga (Valentine, 2009), many Māori researchers have sought to understand and describe this fundamental and esoteric concept. Durie (1994) described one’s wairuatanga as the spiritual Māori dimension of a person’s divine soul that collates the spiritual synergy of the collective. Kake et al. (2020) interprets that our wairua (Māori spirit) provides an ethereal and dynamic link with the gods and ancestors. Kennedy et al. (2015) reflect that wairuatanga is the source of Māori cultural strength and knowledge that completes our identity and is threaded throughout ancestral, present and future Māori realities (Valentine et al., 2017). Pere (1982) described wairua as a means of two complementary, yet opposing, energy forces with physical and spiritual implications. One side of wairua being altruistic, whereas the other as being corrupt and mischevious (Palmer, 2002), and wairuatanga as the act of balancing these forces within our physical and spiritual selves.

When interpreting wairuatanga, we must also consider the principle of mauri (life-force). Mauri is the essence of all life, present in the first breath of life, separating the living from the dead (Palmer, 2002; Tate, 2010). While our bodies exist in the physical world, our mauri and wairua work together to act as two sides of the same coin that form the veil between the physical and spiritual realms, giving life to our physical self and enlivening our spiritual self (Ihimaera, 2004; Valentine et al., 2017).

Wairuatanga can exist as a perceived sensation and lived experience that translates to an everyday practice (Lindsay et al., 2020; Valentine et al., 2017). Our wairua can influence our actions—ensuring caring and respectful relationships between people, the environment, correct Māori practices and whānau—and can, in turn, be affected by the world around us (Kennedy et al., 2015; Lindsay et al., 2020; Valentine, 2009). When our wairua is neglected or subject to abuse through cultural disconnection, social isolation or experiences of trauma, overall health and wellbeing can be affected (Cram et al., 2003; Durie, 1994; Valentine, 2009). A disrupted wairua can, therefore, detrimentally impact our everyday quality of life (Valentine et al., 2017). However, when our wairua is acknowledged and strengthened, our holistic health can be reinforced and improve many aspects of our wellbeing (Durie, 1985). Increased connection to wairua has been found to correlate with positive ageing (Bensemann, 2023). This principle has been speculated to apply to mate wareware, where nourishing our wairua can elevate everyday quality of life and highlight the Indigenous and spiritual tools to improve mate wareware care (Dudley et al., 2019). This wellbeing can be achieved when one’s wairua is consciously nurtured through the actions that acknowledge and nourish our spiritual wellbeing (Lindsay et al., 2020). Although these actions vary for each individual and cannot be postulated, there are likely common aspects of life that can shape wairuatanga. When acknowledged and adhered to, wairuatanga can be strengthened through everyday behaviours.

Wairuatanga is an essential determinant of Māori wellbeing (Cram et al., 2003; Durie, 1985), yet wairuatanga is disregarded within western health disciplines, consequently minimising and ignoring this important contributor to Māori health outcomes (Menzies et al., 2021; Lindsay et al., 2020; Levy & Waitoki, 2015). A significant challenge for western psychology is reconciling Māori realities of wairuatanga with the materialistic reductionist perspective that currently dominates healthcare in Aotearoa (Valentine et al., 2017). It has been speculated that the spiritual healing that wairuatanga practices provide should be included in the prevention, management and treatment of mate wareware (Cram et al., 2003; Menzies et al., 2021). While driving a deeper wairua connection is perceived to support those with mate wareware, the enactment of wairuatanga within the lives of kaumātua has been under-researched, especially for kaumātua with mate wareware (Bensemann, 2023). As wairuatanga is uniquely perceived by each individual, this study examined how wairuatanga manifested in the everyday lives of kaumātua considering a mate wareware diagnosis (Lindsay et al., 2020). Exploring expressions of wairuatanga can influence healthcare approaches to mate wareware care for kaumātua, with the potential to deeply impact Māori wellbeing. The current study analyses responses to the Wairua component of the MANA (Māori Assessment of Neurological Abilities) tool (Dudley et al., 2019).

## Method

The MANA tool is a screening tool developed by a group of Māori-led health clinicians and kaumātua to address the lack of cultural validity seen in western assessment tools currently in use in Aotearoa for detecting mate wareware (Dudley et al., 2019). comprised of a Wairua, Cognitive and Functional component, which combines to form a detailed understanding of the lives of kaumātua and their whānau. The unique Wairua component of the tool targets the wellbeing of Māori spirituality to understand the values and priorities of Māori, build rapport between clinician and patient, and ultimately lead to a more in-depth and valid assessment (Dudley et al., 2019).

The first few questions of the Wairua component focus on the times and places of importance to kaumātua, asking questions such as “*When do you feel well and at peace?*” and “*Where is the place that you feel the mot connected to?*”. The tool leads on to ask about wairuatanga explicitly through questions such as, “*How do you look after your wairua?”* and “*If you feel hōhā* (frustrated, bothered)*, how do you manage this?*”. These are followed by questions surrounding their whānau relationships, including “*How do you ‘get on’ with your whānau?*” and “*How are you involved with your mokopuna* (grandchildren)*?*”. The tool concludes by asking about the changes in their lives as they age, where questions are asked if their “*roles in your whānau or community have changed”* and if they are “*able to manaaki* (look after) *people like you used to?”*. These questions consider many everyday aspects of kaumātua Māori lives, including tūrangawaewae (places of connection), wairuatanga (spiritual understandings), whānau (family experiences) and tuakiritanga (impact on identity) (Menzies et al., 2021).

### Participants

Participants were required to self-identify as Māori and be 55 years or older. In this sample, 61 kaumātua completed the Wairua component of the MANA tool, of which 31 were female and 30 were male, all aged between 60 and 85 years. There were 26 participants from Tāmaki Makaurau, 24 participants from Te Tai Tokerau, and 11 participants from Waikato in Aotearoa New Zealand. All items from the assessment were read aloud to each participant, and the interviewer recorded their summarised responses in short sentences.

### Participant Recruitment

Participant recruitment for this sample of the MANA tool took place between 2020 and 2021. Participants in Tāmaki Makaurau were recruited from the Counties Manukau District Health Board Memory Clinic and a previous Health Research Council (HRC) funded Māori Dementia Feasibility Study where Dr Makarena Dudley was a co-PI (primary investigator). Participants in Te Tai Tokerau were recruited from the patient lists of Whakawhiri Ora Pai in Te Kao and Te Hiku Hauora in Kaitaia. Participants from Waikato were recruited from the Rauawaawa Kaumātua Charitable Trust patient database. A balance of patients was selected – both those who had symptoms suggestive of cognitive impairment and those who did not.

### Interview Process

The participant was scheduled for an interview with a Māori research assistant who had a background in health and was trained to administer the MANA tool. All three components of the MANA tool were administered; however, this study focuses on the Wairua component only. On average, the MANA took one hour to complete. Participants in Tāmaki Makaurau and Kaitaia were given the option to have the interview at their home or a research clinic, and all participants, including their whānau member or caregiver, were given a koha (gift of thanks) to acknowledge their participation in the study.

### Analysis

The data was examined through a Kaupapa Māori research methodology, embracing Māori concepts of aroha (unconditional love and compassion) and manaakitanga (care and guidance) to protect participants’ mana (autonomy and prestige) (Smith, 1999; Walker et al., 2006). Recorded responses were reflexively thematically analysed, with a Māori researcher analysing Māori data and utilising a Māori social constructionist epistemology in doing so. Appropriate to this research on wairua, a critical realist ontology was used, which sought to reflect the concise interpretations of the presence of wairuatanga discussed by kaumātua (Eketone, 2008). Participant responses were organised into four key aspects asked about in the Wairua component, as shown in Table 1 and related to themes of whānau, tūrangawaewae, tuakiritanga and wairuatanga (Menzies et al., 2021). The brief, summarised nature of the data recorded was not able to reflect the significance of these behaviours to kaumātua. As such, the first author selected the responses that reflected the most common stances taken by kaumātua, shaping and refining the coding to form conceptual elements that resonated with the knowledge shared by these kaumātua (Table 1).

**Table 1:** Organisation of questions and participant responses by theme

Themes	Example Questions	Sample Responses
He hononga tangata	How do you get on with your whānau?	*We got a good relationship, we are all good* *Good at times and not so good other times*
Tūrangawaewae	Where does your 'heart' belong? Where is the place you feel most connected to?	*For me, my heart is my family. So where my family are, that's where my heart is* *Owhata—connection of my birth*
Tuakiritanga	Have your roles in your whānau or community changed? → In what ways and how has this made you feel?	*Was involved with marae, but not now* * → That's okay, I still see people now and again* *Yes—not as dependent on me as much* * → Initially it felt lonely, but got use to it*
Mahi mauritau	How do you look after your wairua? If you feel hōhā, how do you manage this?	*Says karakia when he is sad and this helps* *I take a moment—make a concerted effort to be still. Reminding myself to be grateful*

## Results

The personal understandings and unique experiences of wairuatanga in the lives of kaumātua seeking a mate wareware diagnosis revealed four themes. *He hononga tangata* considers the social support available to kaumātua, and *tūrangawaewae* examines the places of importance to kaumātua. *Tuakiritanga* acknowledges the changing aspects of life that impact the identity of kaumātua, and *mahi mauritau* values the spiritual practices that kaumātua undertake.

### He Hononga Tangata

*He hono tangata e kore e motu* is a Māori proverbial saying that values the unbreakable bonds between people, reflecting the significance of relational connections and social support. Within Māori perceptions of the world, whānau is a central social site for kaumātua Māori that consists of relationships intertwined within daily life that are imbued with aroha, manaakitanga and whakapapa (genealogy, ancestry) connections (Butcher & Breheny, 2016; Le Grice et al., 2017). As wairuatanga is fundamentally relational, it has been speculated to be nourished through the actions that retain and strengthen relationships (Le Grice et al., 2017; Valentine et al., 2017).

Within the data, it was common for kaumātua to acknowledge the value of spending time with their whānau. When kaumātua were explicitly asked about their whānau relationships, 48 kaumātua indicated they were on good terms with their whānau. Many mentioned that they lived with or near their whānau and saw or communicated with them regularly. The high presence of whānau interactions indicates that kaumātua value strong relational ties between whānau members. Some kaumātua (13) found their whānau interactions to be so fulfilling that when asked how they choose to enrich their wairua, they respond along the lines of:

*Being around whānau and friends*

Whānau can be fundamental to one’s wairua later in life, likely contributing to the preference of kaumātua to be looked after by whānau rather than be placed in rest homes (Menzies et al., 2021; Butcher & Breheny, 2016; Cullum et al., 2020). Whānau can also support and enrich kaumātua lifestyles and cultivate their future expectations, as evident by the common themes of positive whānau relationships. These thoughts highlight the spiritual value of social support at the end of one’s life, supporting the idea that whānau relationships are essential to both physical and spiritual realms (Butcher & Breheny, 2016; Groot et al., 2011). This support was not universal, and five kaumātua acknowledged that their relationships with their whānau did not always provide comfort and support.

Interestingly, four kaumātua instead chose to acknowledge relationships outside their immediate family, such as their relationships within their neighbourhood, church or marae (Māori meeting place) communities. This supports the idea that kaumātua are social beings who will actively seek social support where they can (Durie, 1999). Some longitudinal studies suggest that a social lifestyle may prevent or delay the onset of mate wareware (Menzies et al., 2021). Participant comments also shed light on the diverse and fluid nature of whānau, which holds different meanings and significance for each kaumātua (Tibble & Ussher, 2012). *He hononga tangata*, therefore, recognises that healthy relationships in the lives of kaumātua may nourish one’s wairua. The relational value of wairuatanga for kaumātua is suggested to be invigorated by strengthening the connection to their nuanced understanding of whānau.

### Tūrangawaewae

The land from which we hail has always been significant to Māori through our whakapapa and history (Mark & Lyons, 2010; Rangiwai, 2018). *Tūrangawaewae* is an inclusive term that refers to the place where one stands, that forms the foundation of one’s worldview, where a person feels comfortable and secure in their rights and can embrace their identity fully (Butcher & Breheny, 2016; Groot et al., 2010; Mark & Lyons, 2010). *Tūrangawaewae* can refer to an ancestral home, a current house or any familiar and warming place, including the physical features of the environment and the social relationships each space provides (Butcher & Breheny, 2016). Wairuatanga has been connected to this innate relationship Māori have with their *tūrangawaewae* (Lindsay et al., 2020; Valentine et al., 2017). When kaumātua were asked about the places they were most connected to, the data revealed three key codes that reflected where kaumātua felt most at peace.

Of the 61 kaumātua in this study, 31 participants referred to their ancestral home as the place they felt genuinely connected. These places included the areas where they were born and raised, the land they are ancestrally linked to, or their marae. As some kaumātua succinctly reported:

*Where I been brought up*

*My marae*

A kaumātua’s connection with their ancestral home, driven by their lived and whakapapa experiences, may preserve a sense of identity in older age by evoking memories and connecting kaumātua with their ancestors (Butcher & Breheny, 2016; Moeke-Maxwell et al., 2013). These ancestral homes may also draw upon traditional Māori stories and history that can connect kaumātua to their culture. One participant used the term *te taunga o te mauri* (a comfort zone or familiar environment) and noted:

*It's like I’m home when I go back there… it’s the spirit of the ancestors, welcoming back*

Twenty one kaumātua also acknowledged the house they were currently residing in to be a source of belonging and contentment, sharing:

*I’m wanted here*

*Happy to be at home here*

These responses indicate that kaumātua have cultivated their current environment to reflect their current needs as ageing people. It can also foster familiarity and comfort, creating a more profound sense of connection to a place of their choosing.

When asked about any intentional actions taken to avoid agitation, it was common for kaumātua to discuss the importance of immersing themselves within their environment. The value of landscape and urban design in supporting health and wellbeing has been previously identified as significant to Māori (Hill, 2021). Seventeen kaumātua indicated that they valued going for walks or tending to their garden to calm and distract themselves from distress. As exemplified by one kaumātua:

*Usually go for walks. Garden is my home*

These actions that value the environment align with the reflections of Durie (1985) and Kennedy et al. (2015), where immersing oneself within the land and environment can energise and rejuvenate one’s wairua.

When asked where their heart belongs, 26 participants said they valued being connected to their whānau rather than any physical place. The confiscation of Māori land during the colonisation of Aotearoa has left some Māori without the opportunity to form a connection with their ancestral land (Simpson et al., 2020; Walker, 2004). It is common for some Māori to consider their whānau to provide the comfort, security and belonging that a particular place can provide. As one kaumātua noted:

*So where my family are, that’s where my heart is*

By repositioning the whānau as the centre of positive memories and fulfilling life, kaumātua have reconstructed what constitutes a *tūrangawaewae* (Durie, 1999). This adaptability also reflects the resilience and determination of kaumātua to create a meaningful life for themselves.

Wairuatanga ties Māori spirituality to the *tūrangawaewae* that kaumātua value in their lives (Valentine et al., 2017). Although one’s mind may not recognise familiar faces or places at times, their wairua may find comfort in this familiarity (Sandberg et al., 2017). Assessing the *tūrangawaewae* of a kaumātua helps to consider the places where wairuatanga may be enriched. This finding has already been recognised by Māori all over Aotearoa, who have built kaumātua flats for their iwi that enable kaumātua to live closer to their ancestral lands and whānau (Ritchie, 1992; Whakatōhea, 2020). However, these flats only provide access to local services and may be limited in the additional health support they can provide kaumātua. This theme highlights the value of the environment that kaumātua immerse themselves in to aid their wellbeing and the surrounding features of their homes (Menzies et al., 2021; Mark & Lyons, 2010).

### Tuakiritanga

Kaumātua are a treasure within society who uphold their people’s status, integrity and traditions (Durie, 1999). *Tuakiritanga* attends to the shaping of identity and the inner being that is an embodiment of their whakapapa and life experiences (Pohatu, 2011). For kaumātua, this considers the persistency of identity through their preference for community roles as they age. Healthy ageing for kaumātua involves personal growth and appropriate ability-related adaptions, which manifest in the suitability of societal positions that kaumātua hold (Hung et al., 2010). As wairuatanga is uniquely perceived by each individual, valuing *tuakiritanga* as kaumātua socially evolve has the potential to cultivate one’s wairua and maintain and strengthen one’s wellbeing (Hill, 2021; Valentine et al., 2017).

When kaumātua were asked about their changing societal roles, the data revealed that 18 kaumātua were accepting of their new positions in society. One kaumātua explained that their decreased responsibilities served them well, providing them with an opportunity to rest. As they stated:

*I used to be … the caretaker, [but now] they put in someone else… I like it because it gave me a rest*

Meanwhile, another kaumātua discussed the significant purpose when becoming more involved with the work on the marae. Summarised in the following quote:

*[I’m] more involved on my marae in [my subtribe]… [it gives me] something to do [with] my life*

Both participants demonstrated how their new roles provided unique opportunities that made their lives meaningful. They also reflected the importance of autonomy in old age and taking on roles aligned with their *tuakiritanga*.

Conversely, kaumātua may become burdened when they adopt roles unsuited to their skills, expertise and identity (Edwards, 2010). This issue is likely to occur if kaumātua battle with declining cognitive ability that restricts one’s ability to act in accordance with their identity. Of the 10 kaumātua who reported feeling unhappy with their new role, a large proportion were concerned with feeling disconnected from their community. Two kaumātua addressed this detachment as follows:

*[My community are] not as dependent on me as much… Initially it felt lonely, but [I] got used to it*

*As I’ve gotten older I became more invisible … [a part of] settling into old age*

Social isolation goes against the traditional habits of kaumātua to work collectively among the people (Awe-Bevan, 2013; Durie, 1999). These social challenges may impact the wellbeing of kaumātua and their ability to live autonomously according to their identity (Hokowhitu et al., 2020). Some kaumātua mentioned this detachment was also associated with the symptoms of mate wareware, as noted:

*Memory deters [her] from being active within [her community]*

These reports of community isolation and lack of autonomy are a concern for the wellbeing of kaumātua, reflecting the challenges kaumātua face in the continuation of western colonisation (Awe-Bevan, 2013; Hokowhitu et al., 2020). Urbanisation has separated some kaumātua from their ancestral lands, marae or genealogical family, and they may not have the same opportunities to contribute to their whanau or marae in their old age (Simpson et al., 2020).

Despite an absence in the literature around the relationship between *tuakiritanga* and wairuatanga, this theme speculates that the alignment of kaumātua realisations of identity with their reality and changes in community roles as they age can influence one’s *tuakiritanga* and wairua (Foster, 2009). Assessing *tuakiritanga* is integral to evaluating factors influencing the wairua of kaumātua and their quality of life as they age.

### Mahi Mauritau

Wairuatanga must be consciously nurtured to avoid neglect (Ihimaera, 2004; Koenig, 2000). *Mahi mauritau* considers the intentional actions taken to enact a state of calmness, which can vary from person to person (He Paiaka Tōtara, 2020). The MANA tool addressed these behaviours within the Wairua component by asking specific questions about how participants looked after their wairua. The data revealed three key areas in kaumātua lives where they engaged in intentional actions to enrich their wairua.

*Mahi mauritau* may refer to the spiritual actions taken to reconnect with the spiritual realm, such as following traditional Māori practices or reciting karakia (Māori prayers) or whakapapa to channel or evoke Māori gods or ancestors (Tate, 2010). These actions provide a connection to the past and can heighten sensitivity to experiences of wairuatanga (Foster, 2009; Lindsay et al., 2020). Twenty one participants said that they care for their wairua by saying karakia, understanding their whakapapa or taking time to think of their ancestors who passed on.

*Do my own karakia*

*Talks to whānau who have passed*

It is common also for kaumātua to find spiritual connections within religious bodies (Andersen, 1940; Klink & Wai, 2019). Spiritual healing may be achieved when people find faith and peace in a higher power (Tate, 2010). Sixteen kaumātua in this study value their connection to religion when acknowledging their wairua through attending church, reading scriptures or praying to a higher power. As discussed by one kaumātua:

*I pray a lot, and I study scriptures, I read up about it*

There are many similarities between both Māori traditional understandings of religion and spiritual connection, and non-traditional understandings. Several kaumātua engaged in both kinds of religious activities, as is the norm for many kaumātua today, indicating the importance of maintaining a connection with spiritual powers for some (Klink & Wai, 2019). When asked how they look after their wairua, one kaumātua discussed both Māori and non-Māori spirituality:

*Family history, karakia, attending church, praying, attending the temple*

Meanwhile, 30 kaumātua in this study chose to look after their wairua through mindful behaviours that did not belong to any religion. When asked about the actions they undertook to look after their wairua and manage their frustrations, many kaumātua found value in engaging in self-care behaviours such as showing kindness to themselves, being grateful for the people in their lives, or meaningfully pondering their emotional state. When asked these questions, some kaumātua responded:

*I take a moment – make a concerted effort to be still. Reminding myself to be grateful*

*I have a little talk to myself. I take this opportunity to acknowledge what’s bothering [me] and calm myself*

These intentional actions eased the minds of kaumātua and settled feelings of unrest, positively impacting their wellbeing (Richards et al., 2010). This finding reflects the significance of daily, mindful activities, similarly as important to kaumātua as religious practices.

As wairuatanga is uniquely perceived by each individual, *mahi mauritau* considers the range of actions kaumātua engage in that relates to their understandings and perceptions of wairuatanga (Lindsay et al., 2020). There was a wide range of behaviours that kaumātua consciously chose to enrich their wairuatanga, reflecting the diverse nature of kaumātua beliefs. It was common for kaumātua to follow western or Māori spiritual beliefs, and many kaumātua engaged in mindful behaviours not associated with any religion that enlightened kaumātua lives. This theme highlights the significance of choosing to nurture wairuatanga and the range of actions that can achieve this (Koenig, 2000).

## Conclusion

This current study emphasises the value of wairuatanga amongst kaumātua being evaluated for the possibility of mate wareware. The themes of *he hononga tangata, tūrangawaewae, tuakiritanga* and *mahi mauritau* each describe a different facet that can describe and enhance wairuatanga.

These facets included the whānau connection, the nourishing role of a spiritual home connection, roles supporting kaumātua identity and practices that kaumātua use to look after their wairua.

This study encourages healthcare systems to consider diverse ways of supporting kaumātua lives throughout mate wareware. Wairua and wairuatanga are currently not well integrated into healthcare systems in Aotearoa, and kaumātua will likely benefit from appropriate cultural support from healthcare providers that value the presence of wairuatanga in kaumātua lives across the four themes identified. This support may include adopting the MANA tool as a mate wareware assessment for Māori kaumātua, enabling mate wareware treatment and future aspirations to be driven by the Wairua, Cognitive and Functional components of the tool.

Future research would be of interest regarding the commonalities and differences within Indigenous communities around the understanding of how the spirit and spirituality supports overall health and wellbeing.

By re-centering Indigenous understandings, kaumātua rights and needs are given appropriate consideration that can help their wellbeing flourish. This study illustrates that kaumātua lives are influenced by everyday behaviours connected to the wellbeing of our wairua, giving significant effect to the potential of our actions to guide our wairua.

## Data Availability

Due to the sensitive nature of the research, supporting data is not available.
